# Cognitive, creative, functional, and clinical symptom improvements in schizophrenia after an integrative cognitive remediation program: a randomized controlled trial

**DOI:** 10.1038/s41537-021-00181-0

**Published:** 2021-10-28

**Authors:** Agurne Sampedro, Javier Peña, Pedro Sánchez, Naroa Ibarretxe-Bilbao, Ainara Gómez-Gastiasoro, Nagore Iriarte-Yoller, Cristóbal Pavón, Mikel Tous-Espelosin, Natalia Ojeda

**Affiliations:** 1 Department of Psychology, Faculty of Health Sciences, University of Deusto, Bilbao, Spain; 2 Refractory Psychosis Unit, Psychiatric Hospital of Álava, Vitoria, Spain; 3 Department of Medicine, Faculty of Health Sciences, University of Deusto, Bilbao, Spain; 4 Department of Physical Education and Sport, Faculty of Education and Sport, University of the Basque Country (UPV/EHU), Vitoria-Gasteiz, Spain

**Keywords:** Schizophrenia, Biomarkers

## Abstract

This study analyzed the effectiveness of an integrative cognitive remediation program (REHACOP) in improving neurocognition, social cognition, creativity, functional outcome, and clinical symptoms in patients with schizophrenia. In addition, possible mediators predicting improvement in functional outcomes were explored. The program combined cognitive remediation with social cognitive training and social and functional skill training over 20 weeks. The sample included 94 patients, 47 in the REHACOP group and 47 in the active control group (occupational activities). Significant differences were found between the two groups in change scores of processing speed, working memory, verbal memory (VM), inhibition, theory of mind, emotion processing (EP), figural creative strengths, functional competence, disorganization, excitement, and primary negative symptoms. A mediational analysis revealed that changes in VM, inhibition, and EP partially explained the effect of cognitive remediation on functional competence improvement. This study provides initial evidence of the effect of integrative cognitive remediation on primary negative symptoms and creativity.

## Introduction

Schizophrenia is considered to be one of the most disabling diseases in the world^1^. People who suffer from it have a high level of dependency in multiple functional domains at very early ages^2,3^, and this functional disability has a major impact on the quality of life of both patients and caregivers or relatives^4^. In spite of the advances in pharmacological treatment, this has not shown to be effective at improving functional outcome^5^. Therefore, functional impairment remains the most significant and challenging treatment target in this disorder.

In an attempt to develop interventions that improve functional disability, many studies have focused on hindering factors^6–9^. Studies have indicated that deficits in neurocognition and social cognition, as well as clinical symptoms, are the most important factors in predicting functional outcome^10–16^. In addition, it has been suggested that other positive personal resources such as creative capacity may have an influence on the functional outcome of people with this disease^9,17,18^. All these findings suggest that interventions aimed at improving these factors could improve functional outcomes. In the last two decades, various meta-analyses have concluded that cognitive remediation is effective in improving not only neurocognition and social cognition, but also clinical symptoms and functional outcomes of people with schizophrenia^19–23^.

It has also been shown that combining different kinds of interventions results in greater improvements than the implementation of cognitive remediation alone^24^. For instance, some studies have combined cognitive remediation with social cognitive training^25–31^ and others have employed a combination of cognitive remediation with social skill training^32^, with functional skill training^33^, or with both social and functional skill training^34,35^. There is also evidence of improvement after cognitive remediation combining cognitive training, social cognitive training, and social and functional skill training^36^. However, the benefits of combining different training forms have only been reported by a heterogeneous and small number of studies. Moreover, some of these studies have suggested that future research should improve several methodological issues that have been raised^25–27,32,36^, including the kind of measures used for the assessment of social cognition, functional outcome, and negative symptoms, as well as the inclusion of an active control group. In addition, owing to the heterogeneity of intervention types, there is no consensus as to how to combine different trainings. Considering that patients with schizophrenia show impairment in numerous domains (e.g., neurocognition, social cognition, social skills, and functional outcome), we expect that combining training in all of these domains would be more effective than, for example, combining only neurocognition and social cognition or neurocognition and social skills training. Furthermore, it is possible that interventions that include social skills training may have an especially beneficial effect on negative symptoms, such as asociality, apathy, or anhedonia^37^.

Another relevant factor that seems to influence functional outcomes among patients with schizophrenia is creativity^9,17^. Several studies have shown that the creative performance of patients with schizophrenia is partly explained by multiple neurocognitive and social cognitive domains^38–41^. In other words, patients with a better capacity in neurocognition and social cognition (e.g., executive functions, processing speed (PS), or theory of mind (ToM) seem to evidence better creative performance. This suggests that patients who improve cognitive functioning could also improve creative capacity, and so if cognitive remediation improves cognition, it is also possible that this improvement may indirectly improve creative ability in this disease. As far as the authors are aware, only one study^42^ to date has analyzed whether cognitive remediation could improve creativity in people with schizophrenia, although as this study lacked a patient control group, more research is needed.

Despite the effectiveness of cognitive remediation in improving functional outcomes, still little is known about the mechanisms through which cognitive remediation proves effective^43^. This is important both for the development of more personalized treatment plans^44^ and for the proper use of healthcare resources^24^. Specifically, research on the cognitive changes associated with functional improvement has tended to be scarce and specific associations are still uncertain^43^. Some of the cognitive domains where changes have been associated with improved functional outcomes include executive functioning^45,46^, working memory (WM)^47^, verbal memory (VM)^15,48^, PS^15,47^, and emotional management^46^. Few studies have explored the cognitive changes related to functional improvement after cognitive remediation combined with other trainings^15,35,46^. For instance, in the study by Eack et al.^46^, improvement in VM, executive functions, and emotional management mediated the relationship between receiving cognitive remediation and functional outcome improvement. In another study^15^, change in VM and PS mediated between receiving cognitive remediation and improvement in functional outcome. Moreover, taking into account the results of other cross-sectional studies that have analyzed predictors of functional outcome^9–13^, it would be expected that improvement in other neurocognitive and social cognitive domains, as well as in clinical symptomatology—mainly negative symptoms— could predict improvement in functional outcome. For example, in the study by Sánchez et al.^35^, improvement in negative symptoms was associated with improvement in functional outcome after cognitive remediation, although no mediation hypothesis was tested.

Taken together, although cognitive remediation has been shown to improve multiple domains in schizophrenia, few studies have encompassed a combination of training in neurocognition, social cognition, social and functional skills. Moreover, some of these studies have reported limitations in the measures employed for social cognition and negative symptoms^25,36^. As far as the authors are aware, no study to date has explored whether integrative cognitive remediation can also improve creativity and primary negative symptoms in people with schizophrenia compared with an active control group.

Therefore, the main aim of this study was to analyze the effectiveness of an integrative group-based cognitive remediation program (REHACOP) that combines training in neurocognition, social cognition, and social and functional skills among patients with schizophrenia in multiple domains: neurocognition, social cognition, creativity, functional outcome, and clinical symptoms. An additional aim was to assess the mechanisms through which functional outcome improves after implementing the integrative cognitive remediation program.

## Results

**Fig. 1:**
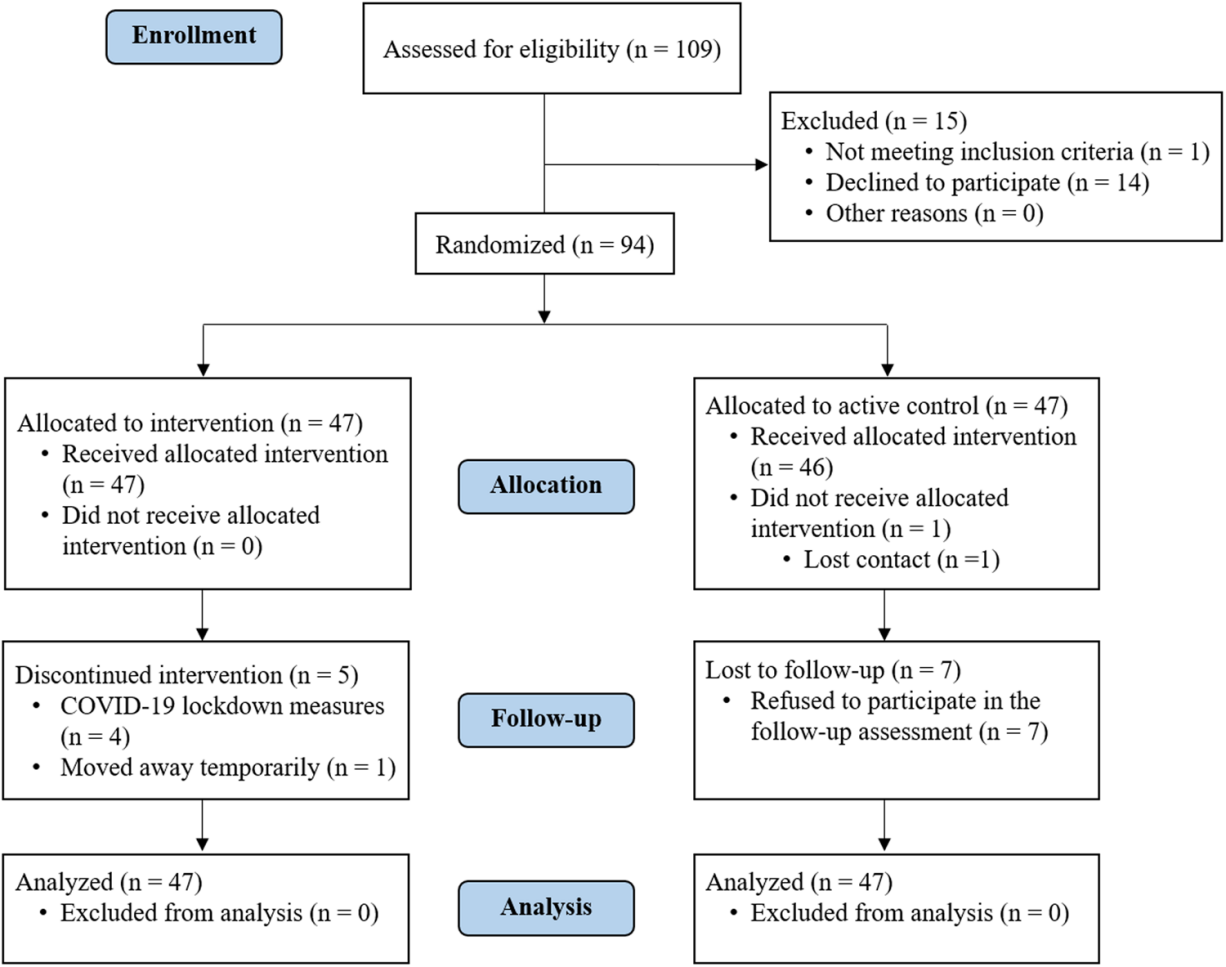
CONSORT flow diagram of study recruitment. CONSORT = Consolidated standards of reporting trials.

Eighty-one patients completed the post-treatment evaluation, resulting in an attrition rate of 13.82% (Fig. 1). Statistical analyses were performed with the 94 randomized patients, following the intention-to-treat (ITT) principle. In addition, per-protocol analyses were also performed only with participants who had completed the post-treatment assessment (Supplementary Tables 1–4). The baseline sociodemographic and clinical characteristics of the REHACOP group and the active control group can be found in Table 1. No significant differences between both groups were found in any of these variables. Statistically significant differences were found between inpatients and outpatients in VM (*F* = 4.034, *p* = 0.048), positive symptoms (*F* = 14.766, *p* < 0.001), primary negative symptoms (*F* = 5.031, *p* = 0.027), disorganization (*F* = 4.847, *p* = 0.030), and excitement (*F* = 4.672, *p* = 0.033) change scores. Specifically, inpatients showed greater improvement in clinical symptoms, whereas outpatients showed greater improvement in VM. The hospitalization status variable was, therefore, introduced as a covariate in the subsequent analyses with these change scores. Regarding satisfaction with the treatment received, the REHACOP group showed greater overall satisfaction compared to the active control group (*U* = 772.00, *p* = 0.010).

**Table 1:** Sociodemographic and clinical characteristics of the sample at baseline.

		REHACOP group (*n* = 47)		Active control group (*n* = 47)			
		Mean *n* (%)	SD	Mean *n* (%)	SD	*t/U/X*^2^	*p*
Age (years)		40.60	10.45	41.43	10.41	0.386	0.701
Education (years)		10.32	2.38	9.87	2.89	0.818	0.416
Gender	Males	41 (87.2%)		37 (78.7%)		1.205	0.272
	Females	6 (12.8%)		10 (21.3%)			
Handedness	Right-handed	39 (80.9%)		38 (83%)		2.613	0.217
	Left-handed	0 (0%)		2 (4.3%)			
	Mixed-handed	9 (19.1%)		6 (12.8%)			
Age of onset (years)		23.92	5.81	22.83	7.36	935.50	0.200
Previous hospitalizations		5.92	6.37	8.56	7.93	865.50	0.070
Hospitalization status	Outpatients	24 (51.1%)		24 (51.1%)		0.000	1.000
	Inpatients	23 (48.9%)		23 (48.9%)			
Medication dosage		501.85	286.64	465.75	188.60	1074.00	0.818
Premorbid IQ		95.13	10.11	93.09	9.98	936.50	0.203

*SD* standard deviation, *t* *t* test, *U* Mann–Whitney *U*, *X*^2^ Chi-squared, Medication dosage refers to chlorpromazine equivalent doses (mg/day).

### Changes in neurocognition, social cognition, and creativity

Significant differences between the REHACOP group and the active control group at baseline were only found in verbal creativity (*U* = 805.00, *p* = 0.023), with the REHACOP group showing higher scores in this domain (Table 2).

**Table 2:** Neurocognitive, creativity, and social cognitive performance in the REHACOP and Active Control groups at baseline and post treatment.

	REHACOP group (*n* = 47)		Active control group (*n* = 47)		Baseline comparison	
	Mean (95% CI)	SD	Mean (95% CI)	SD	*t/U*	*p*
*Neurocognition*						
CF						
Pre	0.08 (−0.14–0.30)	0.74	−0.08 (−0.40–0.19)	1.02	1033.50	0.591
Post	0.15 (−0.07–0.38)	0.74	−0.17 (−0.49–0.13)	1.06		
PS						
Pre	0.15 (−0.12–0.39)	0.90	−0.15 (−0.35–0.06)	0.77	1.676	0.097
Post	0.32 (0.05–0.58)	0.88	−0.33 (−0.56 to −0.11)	0.76		
WM						
Pre	0.16 (−0.08–0.40)	0.86	−0.17 (−0.47–0.15)	1.11	899.00	0.112
Post	0.30 (0.04–0.62)	1.00	−0.34 (−0.58 to −0.08)	0.92		
VM						
Pre	0.02 (−0.23–0.30)	0.92	−0.02 (−0.29–0.28)	0.94	0.171	0.864
Post	0.28 (−0.01–0.58)	1.00	−0.27 (−0.49 to −0.03)	0.81		
Inhibition						
Pre	0.08 (−0.22–0.38)	0.98	−0.08 (−0.33–0.17)	0.87	0.817	0.416
Post	0.31 (0.04–0.59)	0. 92	−0.29 (−0.55 to −0.07)	0.86		
*Social cognition*						
ToM						
Pre	4.55 (3.85–5.25)	2.40	3.70 (2.93–4.47)	2.70	892.50	0.106
Post	5.37 (4.90–5.88)	1.70	3.23 (2.71–3.79)	1.89		
SP						
Pre	9.34 (8.26–10.58)	4.03	8.77 (7.33–10.08)	5.03	1006.50	0.458
Post	10.61 (9.45–11.73)	3.83	10.69 (9.59–11.80)	3.96		
EP						
Pre	14.70 (13.50–15.84)	3.98	14.79 (13.71–15.96)	3.95	1062.50	0.750
Post	16.11 (15.22–16.91)	2.95	14.02 (12.83–15.11)	4.01		
*Creativity*						
Figural creativity						
Pre	50.44 (44.55–56.33)	21.51	44.66 (39.65–50.27)	18.62	1.392	0.167
Post	51.65 (44.51–58.39)	23.30	45.99 (42.17–50.13)	14.34		
Figural creative strengths						
Pre	2.26 (1.67–2.96)	2.32	2.75 (1.95–3.79)	3.23	1034.50	0.591
Post	2.30 (1.59–3.13)	2.53	1.74 (1.19–2.41)	2.17		
Verbal creativity						
Pre	19.89 (17.02–23.42)	10.69	15.14 (13.17–17.14)	7.57	805.00	0.023
Post	20.67 (17.44–23.94)	11.11	17.05 (14.41–19.74)	8.48		

*CF* cognitive flexibility, *PS* processing speed, *WM* working memory, *VM* verbal memory; *ToM* Theory of Mind, *SP* social perception, *EP* emotion processing, *CI* confidence interval, *SD* standard deviation, *t* *t* test, *U* Mann–Whitney *U*. CI was derived from the bootstrap analysis.

Statistically significant differences in change scores between the REHACOP group and the active control group were found in PS, WM, VM, inhibition, ToM, EP, and figural creative strengths (Table 4), in which the REHACOP group improved compared with the active control group. The effect size was medium-large for all these measures, except for figural creative strengths, with a small effect size.

### Changes in clinical symptoms and functional outcome

Significant differences between the REHACOP group and the active control group at baseline were only found in positive symptoms (*t* = −2.14, *p* = 0.035), with the REHACOP group showing higher scores in baseline positive symptoms (Table 3). Analysis of covariance showed statistically significant differences in change scores between the REHACOP group and the active control group in functional competence, primary negative symptoms, disorganization, and excitement (Table 4). The effect size was large for functional outcome, medium for primary negative symptoms and disorganization, and small for excitement.

**Table 3:** Clinical symptoms and functional outcome in the REHACOP and Active Control groups at baseline and post treatment.

	REHACOP group (*n* = 47)		Active control group (*n* = 47)		Baseline comparison	
	Mean (95% CI)	SD	Mean (95% CI)	SD	*t/U*	*p*
*Functional outcome*						
Functional competence						
Pre	63.41 (59.34–67.59)	13.38	60.89 (57.13–64.61)	12.83	0.93	0.353
Post	74.77 (72.14–77.47)	9.33	64.06 (59.65–68.16)	14.45		
Social functioning						
Pre	23.93 (22.71–25.19)	4.49	24.10 (23.08–25.28)	3.70	1073.00	0.809
Post	23.07 (21.66–24.43)	4.67	23.56 (22.61–24.55)	3.44		
Hedonic capacity						
Pre	76.36 (72.80–80.04)	14.05	78.71 (75.63–81.47)	9.55	918.00	0.154
Post	74.86 (71.05–78.28)	12.07	79.04 (75.69–82.23)	11.59		
General self-efficacy						
Pre	60.36 (55.08–66.01)	19.16	58.34 (54.42–62.03)	13.13	1056.50	0.713
Post	64.71 (60.57–69.38)	15.25	58.66 (54.22–63.30)	15.59		
*Clinical symptoms*						
Negative						
Pre	29.57 (25.39–33.74)	13.97	33.80 (29.75–37.62)	13.76	1.148	0.142
Post	22.87 (18.67–27.29)	14.55	32.22 (27.93–36.27)	14.53		
Positive						
Pre	10.35 (9.16–11.59)	4.20	8.66 (7.71–9.66)	3.40	2.143	0.035
Post	8.55 (7.64–9.55)	3.40	8.18 (7.24–9.22)	3.54		
Disorganization						
Pre	7.68 (6.83–8.55)	3.07	7.54 (6.87–8.24)	2.46	0.236	0.814
Post	6.77 (6.02–7.60)	2.84	7.44 (6.80–8.06)	2.25		
Excitement						
Pre	7.62 (6.64–8.65)	3.66	7.10 (6.26–8.07)	3.20	1040.00	0.622
Post	6.37 (5.55–7.27)	2.94	6.87 (5.99–7.92)	3.36		
Depression						
Pre	6.50 (5.85–7.22)	2.46	6.33 (5.71–7.02)	2.30	1053.50	0.697
Post	5.81 (5.10–6.62)	2.51	6.13 (5.49–6.81)	2.32		

*CI* confidence interval, *SD* standard deviation, *t* *t* test, *U* Mann–Whitney *U*. CI was derived from the bootstrap analysis.

**Table 4:** Differences in change scores between the REHACOP and Active Control groups after controlling for baseline scores.

	REHACOP group (*n* = 47)		Active control group (*n* = 47)		ANCOVA for change scores		Effect size
	Mean change score (95% CI)	SE	Mean change score (95% CI)	SE	*F*	*p*	$\eta _p^2$
Neurocognition							
CF	0.10 (−0.11–0.31)	0.10	−0.10 (−0.31–0.10)	0.31	2.10	0.153	0.022
PS	0.20 (0.08–0.33)	0.06	−0.20 (−0.33 to −0.09)	0.06	21.24	0.001	0.190
WM	0.23 (0.01–0.49)	0.12	−0.23 (−0.54–0.04)	0.15	7.27	0.014	0.074
VM	0.27 (0.06–0.48)	0.10	−0.27 (−0.43 to −0.11)	0.08	17.97	0.001	0.166
Inhibition	0.24 (0.03–0.47)	0.11	−0.24 (−0.51 to −0.01)	0.12	9.57	0.006	0.100
Social cognition							
ToM	1.02 (0.45–1.53)	0.27	−0.71 (−1.16 to −0.26)	0.23	37.66	0.001	0.293
SP	1.43 (0.27–2.63)	0.60	1.75 (0.68–2.78)	0.54	0.22	0.618	0.002
EP	1.32 (0.63–2.04)	0.36	−0.73 (−1.64–0.11)	0.44	14.48	0.001	0.137
Creativity							
Figural creativity	1.61 (−3.05–6.68)	2.55	−0.05 (−4.10–4.20)	2.10	0.266	0.586	0.003
Figural creative strengths	−0.14 (−0.71–0.45)	0.30	−0.91 (−1.42 to −0.36)	0.26	4.45	0.034	0.050
Verbal creativity	2.53 (−0.65–5.65)	1.61	−0.04 (−3.05–2.86)	1.55	1.57	0.201	0.020
Functional outcome							
Functional competence	11.72 (9.46–13.96)	1.14	2.86 (0.49–5.19)	1.17	30.85	0.001	0.253
Social functioning	−0.99 (−2.05–0.08)	0.56	−0.55 (−1.33–0.21)	0.40	0.45	0.511	0.005
Hedonic capacity	−2.10 (−4.74–0.34)	1.29	0.63 (−1.63–2.89)	1.11	2.66	0.123	0.028
General self-efficacy	5.12 (0.41–9.95)	2.42	−0.79 (−7.22–4.97)	3.13	3.25	0.075	0.036
Clinical symptoms							
Negative	−6.83 (−9.18 to −4.58)	1.24	−1.60 (−3.60–0.12)	0.93	10.95	0.003	0.108
Positive	−1.56 (−2.29 to −0.80)	0.38	−0.79 (−1.56 to −0.08)	0.38	2.606	0.110	0.028
Disorganization	−0.97 (−1.48 to −0.49)	0.24	−0.13 (−0.47–0.25)	0.18	8.513	0.007	0.086
Excitement	−1.20 (−1.74 to −0.65)	0.27	−0.24 (−0.98–0.49)	0.38	4.606	0.041	0.049
Depression	−0.71 (−1.32–0.04)	0.34	−0.20 (−0.77–0.37)	0.29	1.584	0.227	0.017

*CF* cognitive flexibility, *PS* processing speed, *WM* working memory, *VM* verbal memory, *ToM* Theory of Mind; *SP* social perception, *EP* emotion processing, *CI* confidence interval, *SE* standard error, *ANCOVA* analysis of covariance, $\eta _p^2$ partial eta squared, Change scores post-treatment score−pre-treatment score. Means for change scores are adjusted for the effect of the baseline score. CI and SE for change scores were derived from the bootstrap analysis. Significance levels were determined using *F* tests based on the bootstrap SE estimate for that comparison, rather than using a pooled SE estimate.

### Mediational model explaining improvement in functional outcome

A mediational analysis was performed on the change score of functional competence. A model with a path analysis was estimated which included only those mediating variables that had had a significant correlation with the change in functional competence: VM, inhibition, EP, and primary negative symptoms. The independent variable was the group (REHACOP group vs active control group). Based on previous literature^49–52^, the baseline score of functional competence as well as age and IQ were included to control their possible influence. The model had a good fit, SB *χ*^2^ (7, *N* = 94) = 2.670, Comparative Fit Index (CFI) = 1.000, non-normed fit index (NNFI) = 1.000, and standard residual mean square root (SRMR) = 0.033.

**Fig. 2:**
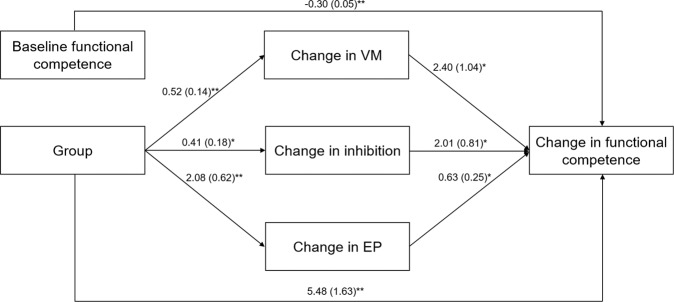
Model of mediation explaining improvement in functional competence after the integrative cognitive remediation through changes in VM, inhibition, and EP. Given values are non-standardized coefficients with standard errors in parentheses. *p ≤ 0.05, **p ≤ 0.001.

The covariance between the group and baseline score for functional competence, the covariance between the mediating variables, the paths from age and IQ to the mediating and outcome variables, and the path from change score in primary negative symptoms to the outcome variable were not statistically significant. Therefore, in order to obtain a more parsimonious model, a new model was estimated that included only the significant paths, omitting those variables that were not associated with mediators or outcome variables (age, IQ, and change score in primary negative symptoms). The final model obtained is shown in Fig. 2. As can be seen, group (for the REHACOP group) was positively related to changes in VM, inhibition, and EP, and these three mediating variables were positively associated with change in functional competence. In addition, the group was directly and positively associated with a change in functional competence. Finally, baseline functional competence was also directly and negatively associated with a change in functional competence, indicating that a lower baseline score was related to greater change in functional outcome after the intervention. The model also had a good fit, SB *χ*^*2*^ (7, *N* = 94) = 4.282, CFI = 1.00, NNFI = 1.00, and SRMR = 0.052. Next, the significance of the mediational paths was examined via 5,000 bootstrapping samples. The results revealed that change in VM (1.260; 95% confidence interval [1.260, 1.296]), change in inhibition (0.854; 95% confidence interval [0.838, 0.870]), and change in EP (1.306; 95% confidence interval [1.285, 1.326]) acted as mediating variables between group and change in functional competence.

## Discussion

The first aim of this study was to analyze the effectiveness of an integrative group-based cognitive remediation program (REHACOP) that combined training in neurocognition, social cognition, as well as social and functional skills among patients with schizophrenia. In line with previous studies carried out with integrative interventions combining training in neurocognition with social cognition and/or social and functional skills^25,27,28,31,34–36^, patients from the REHACOP group showed significantly greater improvement than patients from the active control group in neurocognition, social cognition, creativity, functional outcome, and clinical symptoms.

As expected and consistent with previous research^25,27,28,31,34–36^, the REHACOP group showed improvements in numerous neurocognitive and social cognitive domains. In particular, very large effect sizes were found in PS, VM, ToM, and emotion processing (EP). The significant results obtained in social cognition are particularly interesting, since they reinforce the findings of previous studies that used different types of measures^25,31,36^ (e.g., paper-and-pencil tasks instead of videos). Contrary to some previous studies, no significant results were found for cognitive flexibility (CF)^25,53,54^ and social perception (SP)^36,55^. Nevertheless, SP has received little attention in studies, and those that have assessed it have done so in different ways.

Regarding creativity, although this capacity was not directly trained in the intervention, we hypothesized that it would be indirectly improved through enhancements in social cognition and neurocognition^38–41^. In fact, some of the abilities trained in the REHACOP included novel problem solving and CF, which is closely related to creativity^38,40^. Significant differences between groups were found only in the change scores of figural creative strengths. Although the REHACOP group showed only a very small improvement in figural creative strengths at post-treatment, the active control group showed a decrease in this creativity variable. The fact that significant results were not found in the other creativity domains could be partly due to the kind of occupational activities that the active control group performed (e.g., handicrafts, painting, and music), which are closely related to creativity. The lack of research makes it difficult to compare these results with other studies. Kiritsis^42^ found an improvement in creativity after cognitive remediation in patients with schizophrenia, but their results were not compared with a patient control group.

With respect to functional outcome and in line with previous studies carried out with integrative cognitive interventions^28,34,36^, the REHACOP group showed significantly higher change scores in functional competence, with a very large effect size. Significant results were not found for other functional outcome measures, which could be partly due to the smaller sample size of these measures. In addition, the COVID-19 pandemic may have influenced these results, as some of the social activities that were assessed may have been undermined by lockdown measures, which were especially restrictive when sessions with several intervention groups were held. Other studies that have applied a combined intervention did not find significant results in functional outcome measured through similar dimensions, such as social functioning and hedonic capacity^29,30^, and so cognitive remediation could benefit from also including direct intervention in social functioning (pro-social activities, recreation, etc.) and hedonic capacity.

With regard to clinical symptoms, the REHACOP group showed a significant reduction in excitement, disorganization, and primary negative symptoms compared to the active control group, with small and medium effect sizes. The improvement in disorganization and negative symptoms in the REHACOP group was in line with some previous studies^25,31,35,36^, but not with others^26,29,34^. Nevertheless, it is worth mentioning that these previous studies did not assess primary negative symptoms separately, but rather, primary and secondary symptoms together. The improvement in primary negative symptoms found in this study is an important result that indicates that the effects of cognitive remediation can be generalized to other domains that are not directly trained. It may be possible for the inclusion of training in social skills to have a particular beneficial effect on improving these symptoms^37^. However, since different cognitive remediation interventions were not compared in this study, this idea should be considered with caution. There is less evidence about the improvement in excitement after cognitive remediation^56^. The lack of improvement in positive symptoms was congruent with previous studies^26,29,34–36^.

Interestingly, several differences were found between outpatients and inpatients in VM and several clinical symptom change scores. Whereas outpatients benefited more in terms of VM, inpatients showed a greater improvement in clinical symptoms. These differences in change scores cannot be attributed to the baseline scores or to the medication dosage, since baseline significant differences between outpatients and inpatients were only found in excitement and this was entered as a covariate. Regarding the greater improvement in clinical symptoms of inpatients, this may be partly attributed to the hospital care routine that may have contributed to the stabilization of symptoms (e.g., defined schedules for daily activities, healthier lifestyle habits, personal care, etc.)^57^, in contrast to some outpatients that may have a more disorganized daily routine. The greater improvement found in outpatients in VM is in contrast with results from Cella et al.^22^, a meta-analysis in which it is suggested that the inpatient may gain greater benefit in cognition compared with community samples. Nevertheless, the sample size of this study does not allow us to draw conclusions as to the influence of hospitalization status.

The second objective of this study was to explore the mediating mechanisms through which integrative cognitive remediation improved functional outcomes. In this study, changes in VM, inhibition, and EP partially mediated the association between receiving the intervention and improvement in functional competence. In addition, lower baseline functional competence scores were related to a greater change in this domain. The finding that the group variable in the mediational model continued to have a direct association with change in functional competence was expected since the REHACOP group included training in functional skills. The few studies that have assessed the association between baseline functioning and functional improvement after cognitive remediation in schizophrenia^33,51,52,58,59^ have multiple methodological differences (e.g., measurement of functional outcome or type of intervention), which does not allow suitable comparison of results. For instance, among those studies in which a performance-based measure of functional competence was used (e.g., UPSA), Twamley et al.^52^ found lower baseline functional competence to be related to greater changes in functional competence. However, this study included patients with other disorders in addition to schizophrenia, such as schizoaffective disorder, and the intervention involved compensatory cognitive training rather than a drill and practice cognitive remediation. In contrast, Kurtz^51^ found that higher baseline functional competence was related to greater functional competence at post-treatment assessment, although this study also included both patients with schizophrenia and schizoaffective disorder who had engaged in computerized cognitive remediation or computer-skills training. Given the great disparity found among these studies, more research is needed^44^.

Regarding the predicting role of cognitive changes, previous studies have also found changes in VM^15,46,48^ and executive functions^45,46^ to predict change in functional outcome. Similar to EP, Eack et al.^46^ found improvement in emotion management to influence change in functional outcome. Interestingly, in the study by Eack et al.^46^, changes in VM, executive functions, and emotional management were also the only domains that predicted change in functional outcome. In the present study, change scores in primary negative symptoms did not mediate improvement in functional competence, which was in line with the limited literature available^15^. In contrast, in the study by Sánchez et al.^35^, improvement in negative symptoms was associated with improvement in functional outcome after cognitive remediation, although no mediation hypothesis was tested. As a whole, these results suggest that cognitive remediation should emphasize training in VM, executive functions, and EP. Nevertheless, findings of this topic are heterogeneous among studies and therefore, more research is needed before conclusions can be drawn. For instance, in the study by Peña et al.^15^, executive functions, which were assessed using the same measure as in this study, failed to explain the functional improvement. Nor did EP, although this was measured using a different instrument^15^.

Several limitations should be considered in this study. First, the sample size may have limited the significance of some results, especially for some functional outcome measures which included a smaller sample size, and therefore, more data had to be imputed. Second, the sample was skewed towards men, although there were no sex differences between both groups. Third, the COVID-19 pandemic caused several patients to drop out and some intervention sessions had to be temporarily suspended, although they were successfully resumed at a later stage. Fourth, the power analysis was only conducted for neurocognitive domains and thus, the rest of the domains should be considered as exploratory outcomes. Fifth, patients were not blind to the treatment condition received, although they were instructed not to mention what type of treatment they would receive or had received during evaluations, to ensure masking of the evaluators. The fact that patients from the active control group were aware of being in the control condition may have influenced the results. Therefore, future studies should try to ensure all participants remain blind to the treatment condition. However, it is worth mentioning that this study ensured that the active group received as much social interaction as the REHACOP group.

Bearing these limitations in mind, these findings support previous evidence of the effectiveness of integrative cognitive remediation in improving multiple domains, while overcoming some methodological issues raised in previous studies^25–27,32,36^. Moreover, the study provides preliminary relevant evidence of the —somewhat slight effectiveness—of integrative cognitive remediation in a creative capacity. In addition, this study shows that integrative cognitive remediation is effective at improving primary negative symptoms. All these results have relevant implications for the design of treatment plans for patients with schizophrenia. A major implication is that these findings provide strong evidence to suggest that integrative cognitive remediation combining training in neurocognition, social cognition, and social and functional skills should be included as a key intervention in the treatment plans for patients with schizophrenia, as this intervention is even capable of ameliorating the primary negative symptoms that are so resistant to pharmacological treatment. A second implication is linked to the future lines of research that these results open up. Considering that creativity is a key factor in the functional outcome of these patients^9^ and that cognitive remediation has been shown to produce a slight beneficial effect on it, a second step would be to study whether including training in creativity in combination with this integrative cognitive intervention could improve creativity, and in turn increase benefits in terms of functional outcome. Furthermore, it would be interesting for future studies to analyze whether creativity is a valued outcome in patients with schizophrenia. Moreover, future studies could start including training not only in creativity but also in other positive personal resources, such as humor^60^ or resilience^12^, within integrative cognitive remediation, to see whether these abilities produce greater benefits. In fact, the inclusion of training in these positive resources could increase motivation and adherence to treatment. Another implication is that findings from this study, in line with those of Eack et al.^46^, suggest that cognitive remediation should reinforce training in VM, executive functions, and EP with a view to obtaining a greater benefit in terms of functional competence. This reinforces the idea that interventions benefit from combining training in both neurocognition and social cognition. Finally, future studies might benefit from separately analyzing the effect of cognitive remediation in outpatients and inpatients in order to gain a more-detailed understanding of the type of patients who benefit most.

## Methods

### Participants

The sample consisted of 94 patients diagnosed with schizophrenia who were recruited from the Psychiatric Hospital of Álava and the Mental Health Network in Álava (Spain). All patients had been diagnosed with schizophrenia according to the Diagnostic and Statistical Manual of Mental Disorders, Fifth Edition (DSM-5)^61^. In addition, all patients showed cognitive impairment. The exclusion criteria were as follows: (a) clinical instability according to the criteria of Csernansky et al.^62^; (b) relevant modifications in the antipsychotic treatment in the previous 3 months due to an increase in the severity of clinical symptoms; (c) cognitive impairment caused by another disease; (d) diagnosis of an active major affective disorder; (e) being in another specific cognitive remediation program; and (f) Substance Use Disorder (DSM-5)^61^ within the 3 months before screening. The sample included both outpatients and inpatients. The inpatient study sample consisted of patients hospitalized at a psychiatric rehabilitation unit at the Psychiatric Hospital of Álava who were anticipating imminent hospital discharge to the community setting.

### Procedure

The sample size was determined through an a priori power analysis, based on a previous study on REHACOP^36^, which used the G*Power 3 software^63^. In order to obtain an effect size of *d* = 0.77 to see group differences in neurocognition, with 90% power and a 5% level of significance, a sample size of 76 subjects, 38 in each group, was deemed adequate. The rest of the outcome measures were considered to be exploratory.

The study design was a parallel-group randomized trial, forming part of a larger project that has been later expanded to include an additional arm (a group receiving intense physical exercise). The cognitive remediation arm has been completed, while the physical exercise arm is ongoing. Therefore, in the present study, only the cognitive remediation group and the active control group were included. The participants were randomly assigned to each group (Fig. 1), this being done via an online computer-generated randomization system (http://www.randomization.org). Randomization was performed by a Study Coordinator who was not involved in the assessment, study interventions, or statistical analyses. All participants underwent a neuropsychological and psychiatric assessment at pre-treatment and at a 5-month follow-up, with post-treatment evaluation being conducted within the first three weeks following the end of the intervention. All raters were blind to the experimental treatment condition and had no other role in the study (they had no clinical responsibility for the participants during the completion of the study and were not involved in administering the cognitive remediation or occupational activities) that could undermine the trial blinding. Moreover, participants were instructed not to mention what type of treatment they would receive or had received during evaluations.

The study protocol received the approval of the Clinical Research Ethics Committees of the Autonomous Region of the Basque Country in Spain (PI2017044), and the project is registered with clinicaltrials.gov (NCT03509597). All patients participated voluntarily, providing written informed consent to take part, and did not receive any monetary reward for participating in the project.

### Measures

The evaluation of neurocognition included measurement of the following domains: CF, PS, WM, VM, and inhibition. All the scores were converted into Z scores based on the study sample and some scores were adjusted to make higher scores indicate better cognitive performance. A composite score was calculated using the number of categories completed and the number of perseverative errors obtained using the Modified Wisconsin Card Sorting Test^64^ for assessment of CF (Cronbach’s alpha = 0.74). PS was measured by the Symbol-Coding from the Wechsler Adult Intelligence Scale-III (WAIS-III)^65^ and the Stroop Color and Word Test (SCWT)^66^ (Word and Color scores) (Cronbach’s alpha = 0.80). The Backward Digit Span from the WAIS-III^65^ was used for assessment of WM, whereas VM was measured using a composite score obtained from the three learning trials and the delayed recall trial in accordance with the Hopkins Verbal Learning Test Revised (version 2 at baseline and 4 at post treatment)^67^ (Cronbach’s alpha = 0.84). Finally, the SCWT^66^ (Word-Color and Interference scores) was used for inhibition (Cronbach’s alpha = 0.84).

Social cognition was measured through ToM, SP, and EP. The Happé Test “Strange Stories Task”^68^ was used for the assessment of ToM (four different stories at baseline and follow-up). SP was measured through the Social Attribution Task-Multiple Choice (version 2 at baseline and 1 at post-treatment)^69^, a video of a social drama enacted by geometric figures. EP was evaluated through the Spanish adaptation of the Bell Lysaker Emotion Recognition Test (version 2 at baseline and 1 at post-treatment)^70^, a video showing an actor portraying different effects.

Creativity was evaluated using two subtests from the Torrance Test of Creative Thinking^71,72^ (version A at baseline and version B at post-treatment). Figural creativity was assessed with the Picture Completion subtest. The total figural creativity score was calculated by adding scores for originality, elaboration, fluency, resistance to premature closure, the abstractness of titles, and flexibility. In addition, a figural creative strengths score was obtained according to the manual^72^. The Unusual Uses subtest was used for the assessment of verbal creativity. Total verbal creativity score was calculated by adding scores for the originality, fluency, and flexibility dimensions. A more-detailed explanation of these tests has been provided elsewhere^39^.

The assessment of functional outcome included the following tests. Functional competence was measured by the Spanish Version of the University of California, San Diego, Performance-Based Skills Assessment^73^, assessing the performance of everyday activities in different areas. Social functioning was evaluated by means of the short Spanish version of the Social Functioning Scale^74^, whereas the Spanish adaptation of the Anticipatory and Consummatory Interpersonal Pleasure Scale-Adult version^75^ was used to assess hedonic capacity for social interactions. General self-efficacy was measured using the Spanish adaptation of the General Self-Efficacy Scale^76^. Sample size with pre-treatment assessment on the Social Functioning Scale, the Anticipatory and Consummatory Interpersonal Pleasure Scale-Adult version, and the General Self-Efficacy Scale was smaller (*n* = 42 for the REHACOP group and *n* = 24 for the active control group).

Regarding clinical symptoms, the Positive and Negative Syndrome Scale^77^ was employed to measure positive symptoms, disorganization, excitement, and depression, according to the five-factor solution proposed by Wallwork et al.^78^. As recommended by the NIMH-MATRICS Consensus Statement on Negative Symptoms^79,80^, primary negative symptoms were assessed using the Brief Negative Symptom Scale^81^.

Satisfaction with the received treatment was assessed through the Spanish adaptation of the Consumer Reports Effectiveness Scale^82^.

Premorbid IQ was measured by the Word Accentuation Test^83^, a Spanish version of the National Adult Reading Test^84^. Premorbid IQ was estimated using raw scores that were converted using the full-scale IQ of Gomar et al.^85^.

Handedness was assessed by means of the Edinburgh Handedness Inventory^86^. The following formula was used to estimate handedness consistency: right−left/right+left.

### Intervention

REHACOP is a group-based integrative cognitive remediation program that combines training in neurocognition, social cognition, social skills, and functional skills^87^. It integrates top–down and bottom–up strategies and includes paper-and-pencil tasks, active group discussions, and role-playing. REHACOP is highly structured, which minimizes the effect of being administered by different therapists. This intervention program includes up to 300 different tasks that are divided into different units and subtypes of abilities. Tasks within each unit are hierarchically ordered according to a subtype of abilities and levels of complexity to ensure a gradual increase in cognitive demand (further explanation about REHACOP is provided elsewhere^35^).

In this study, sessions were arranged for nine groups of between four and eight patients each, at various centers which formed part of the Mental Health Network in Álava (the Psychiatric Hospital of Álava, the Association of Relative and Patients with Mental Illness from Ayala, and the Community Rehabilitation Service Center). The clinical team who conducted the intervention was trained in administering REHACOP and used the same materials and instructions in all the groups, with the 60-minute sessions being held 3 days a week over 20 weeks. The REHACOP intervention group trained the following units: Attention unit (4 weeks), with training in selective, sustained, alternating, and divided attention; Learning and Memory unit (4 weeks), including visual and verbal learning, recall, recognition memory, WM, and compensatory strategies; Language unit (3 weeks) focused on syntax, vocabulary, grammar, verbal comprehension, verbal fluency, and abstract language; Executive Functions unit (3 weeks), including cognitive and objective planning, novel problem solving, CF, reasoning, categorization, and conceptualization; Social Cognition unit (3 weeks), with training in EP, social reasoning, moral dilemmas, and ToM; Social Skills unit (2 weeks); and Functional Skills unit (1 week), including activities involved in daily living. In addition, several tasks were timed to train PS throughout the first four units.

The active control group carried out occupational group activities (gardening, sewing, handicrafts, painting, and music) with the same duration and frequency as the REHACOP group. In addition, as part of the standard treatment, patients from both the experimental and active control groups received psychoeducation sessions.

If a patient missed one or more sessions for various reasons (e.g., vacation or leave), they received individual training in the contents that had been trained in the group session or alternatively, received feedback through homework afterward. This allowed the patient to meet the objectives of all the training sessions missed. The patient then rejoined the experimental group. Therefore, patients made up all the missed sessions. Moreover, the maximum number of sessions missed permitted was six (2 weeks). Patients who stopped attending sessions and did not return were considered dropouts (please see Fig. 1). Owing to the COVID-19 pandemic, several experimental (*n* = 13) and active control (*n* = 2) groups were temporarily discontinued at the beginning of the interventions. Therefore, individual booster sessions were conducted for 2 weeks before intervention groups were resumed.

### Data analyses

IBM SPSS version 26.0 (SPSS Inc., Chicago, USA) was used for statistical analyses. Statistical analyses were carried out according to the ITT principle (*N* = 94). In addition, per-protocol analyses were also performed (Supplementary Tables 1–4) only with those participants who had completed the post-treatment assessment (*N* = 81). The expectation-maximization algorithm was used to impute missing values. The Little’s Missing Completely at Random test showed that the missing data were missing completely at random (*X*^*2*^ [6412] = 2800.45, *p* = 1.000). The Shapiro–Wilk test was used to test data for normality. Differences between groups in terms of sociodemographic, cognitive, creative, functional outcome, and clinical variables at baseline were assessed by a two-tailed independent *t* test or Mann–Whitney *U* test. Differences between groups in categorical data were analyzed using the Chi-squared (*X*^*2*^) test.

An analysis of covariance was used to analyze the effectiveness of the cognitive remediation, with baseline scores being entered as covariates. Specifically, analysis of covariance was applied to compare change scores (post-treatment−baseline) between the REHACOP group and the active control group on each of the cognitive, creative, functional outcome, and clinical variables, controlling baseline scores. A bootstrapping procedure^88^ was performed (1000 samples) to obtain adjusted mean differences in change scores, using the Bonferroni adjustment. Effect sizes were calculated using partial eta squared (${\eta_p^2}$) and this was considered small (0.01), medium (0.06), or large (0.14)0.^89^ The significance level was set at 0.05, and all tests were two-tailed.

In order to analyze predictors of functional outcome, Spearman’s Rho and Pearson’s *r* correlation analyses were first carried out between those neurocognitive, social cognitive, creative, and clinical change variables and functional outcome variables that had shown improvement after the intervention. LISREL 9.2^90^ was then used to perform a path analysis which was conducted to assess the mediating role of these change scores in the relationship between receiving integrative cognitive remediation (vs active control group) and improvement in functional outcome. The robust maximum likelihood method was used, which requires an estimate of the asymptotic covariance matrix of the variances and covariates of the sample, including the scaled *χ*^2^ Satorra-Bentler index (SB *χ*^2^). The CFI, NNFI, and SRMR were used to evaluate the goodness of fit of the model. According to Hu and Bentler (1999), CFI and NNFI values higher than 0.90 and SRMR values smaller than 0.08 indicate a good fit^91^.

### Reporting summary

Further information on research design is available in the Nature Research Reporting Summary linked to this article.

## Supplementary information

Supplementary Information

Reporting Summary

## Data Availability

The data that support the findings of this study are available from the corresponding author on reasonable request. The data are not publicly available due to them containing information that could compromise research participant privacy or consent.

## References

[1] Vos T (2017). Global, regional, and national incidence, prevalence, and years lived with disability for 328 diseases and injuries for 195 countries, 1990-2016: a systematic analysis for the Global Burden of Disease Study 2016. Lancet.

[2] Conus P, Cotton S, Schimmelmann BG, McGorry PD, Lambert M (2007). The first-episode psychosis outcome study: premorbid and baseline characteristics of an epidemiological cohort of 661 first-episode psychosis patients. Early Interv. Psychiatry.

[3] Harvey PD (2014). Disability in schizophrenia: contributing factors and validated assessments. J. Clin. Psychiatry.

[4] Fleischhacker WW (2014). Schizophrenia-Time to commit to policy change. Schizophr. Bull..

[5] Green MF, Horan WP, Lee J (2019). Nonsocial and social cognition in schizophrenia: current evidence and future directions. World Psychiatry.

[6] Galderisi S (2014). The influence of illness-related variables, personal resources and context-related factors on real-life functioning of people with schizophrenia. World Psychiatry.

[7] Bowie CR (2008). Predicting schizophrenia patients’ real-world behavior with specific neuropsychological and functional capacity measures. Biol. Psychiatry.

[8] Green MF (2016). Impact of cognitive and social cognitive impairment on functional outcomes in patients with schizophrenia. J. Clin. Psychiatry.

[9] Sampedro A (2021). The impact of creativity on functional outcome in schizophrenia: a mediational model. npj Schizophr..

[10] Strassnig MT (2015). Determinants of different aspects of everyday outcome in schizophrenia: the roles of negative symptoms, cognition, and functional capacity. Schizophr. Res..

[11] Ojeda N (2019). An outcome prediction model for schizophrenia: a structural equation modelling approach. Rev. Psiquiatr. Salud Ment..

[12] Galderisi SG (2014). The influence of illness-related variables, personal resources and context-related factors on real-life functioning of people with schizophrenia. World Psychiatry.

[13] Fervaha G, Foussias G, Agid O, Remington G (2014). Impact of primary negative symptoms on functional outcomes in schizophrenia. Eur. Psychiatry.

[14] Fu S, Czajkowski N, Rund BR, Torgalsbøen AK (2017). The relationship between level of cognitive impairments and functional outcome trajectories in first-episode schizophrenia. Schizophr. Res..

[15] Peña J (2018). Mechanisms of functional improvement through cognitive rehabilitation in schizophrenia. J. Psychiatr. Res..

[16] Green MF, Kern RS, Braff DL, Mintz J (2000). Neurocognitive deficits and functional outcome in schizophrenia: are we measuring the ‘right stuff’?. Schizophr. Bull..

[17] Nemoto T, Kashima H, Mizuno M (2007). Contribution of divergent thinking to community functioning in schizophrenia. Prog. Neuro-Psychopharmacol. Biol. Psychiatry.

[18] Nemoto T (2009). Cognitive training for divergent thinking in schizophrenia: a pilot study. Prog. Neuropsychopharmacol. Biol. Psychiatry.

[19] McGurk SR, Twamley EW, Sitzer DI, McHugo GJ, Mueser KT (2007). A meta-analysis of cognitive remediation in schizophrenia. Am. J. Psychiatry.

[20] Wykes T, Huddy V, Cellard C, McGurk SR, Czobor P (2011). A meta-analysis of cognitive remediation for schizophrenia: Methodology and effect sizes. Am. J. Psychiatry.

[21] Revell ER, Neill JC, Harte M, Khan Z, Drake RJ (2015). A systematic review and meta-analysis of cognitive remediation in early schizophrenia. Schizophr. Res.

[22] Cella M (2020). Cognitive remediation for inpatients with psychosis: a systematic review and meta-analysis. Psychol. Med..

[23] Cella M, Preti A, Edwards C, Dow T, Wykes T (2017). Cognitive remediation for negative symptoms of schizophrenia: a network meta-analysis. Clin. Psychol. Rev..

[24] Cella M, Reeder C, Wykes T (2015). Cognitive remediation in schizophrenia — now it is really getting personal. Curr. Opin. Behav. Sci..

[25] Mueller DR, Schmidt SJ, Roder V (2015). One-year randomized controlled trial and follow-up of integrated neurocognitive therapy for schizophrenia outpatients. Schizophr. Bull..

[26] Lindenmayer JP (2018). Does social cognition training augment response to computer-assisted cognitive remediation for schizophrenia?. Schizophr. Res..

[27] Bechi M (2015). Combined social cognitive and neurocognitive rehabilitation strategies in schizophrenia: neuropsychological and psychopathological influences on Theory of Mind improvement. Psychol. Med..

[28] Fisher M (2017). Supplementing intensive targeted computerized cognitive training with social cognitive exercises for people with schizophrenia: an interim report. Psychiatr. Rehabil. J..

[29] Fernandez-Gonzalo S (2015). A new computerized cognitive and social cognition training specifically designed for patients with schizophrenia/schizoaffective disorder in early stages of illness: a pilot study. Psychiatry Res..

[30] Hooker CI (2012). Neural activity during emotion recognition after combined cognitive plus social-cognitive training in schizophrenia. Schizophr. Res.

[31] Eack SM (2009). Cognitive enhancement therapy for early course schizophrenia: effects of a two-year randomized controlled trial. Psychiatr. Serv..

[32] Galderisi S (2010). Social skills and neurocognitive individualized training in schizophrenia: comparison with structured leisure activities. Eur. Arch. Psychiatry Clin. Neurosci..

[33] Bell M, Zito W, Greig T, Wexler BE (2008). Neurocognitive enhancement therapy and competitive employment in schizophrenia: Effects on clients with poor community functioning. Am. J. Psychiatr. Rehabil..

[34] Bowie CR, Mcgurk SR, Mausbach B, Patterson TL, Harvey PD (2012). Combined cognitive remediation and functional skills training for schizophrenia: effects on cognition, functional competence, and real-world behavior. Am. J. Psychiatry.

[35] Sánchez P (2014). Improvements in negative symptoms and functional outcome after a new generation cognitive remediation program: a randomized controlled trial. Schizophr. Bull..

[36] Peña J (2016). Combining social cognitive treatment, cognitive remediation, and functional skills training in schizophrenia: a randomized controlled trial. npj Schizophr..

[37] Granholm E, Holden J, Link PC, Mcquaid JR (2014). Randomized clinical trial of cognitive behavioral social skills training for schizophrenia: improvement in functioning and experiential negative symptoms. J. Consult. Clin. Psychol..

[38] Sampedro A (2019). Mediating role of cognition and social cognition on creativity among patients with schizophrenia and healthy controls: revisiting the shared vulnerability model. Psychiatry Clin. Neurosci..

[39] Sampedro A (2020). Neurocognitive, social cognitive, and clinical predictors of creativity in schizophrenia. J. Psychiatr. Res..

[40] Abraham A, Windmann S, McKenna P, Güntürkün O (2007). Creative thinking in schizophrenia: the role of executive dysfunction and symptom severity. Cogn. Neuropsychiatry.

[41] Jaracz J, Patrzała A, Rybakowski JK (2012). Creative thinking deficits in patients with schizophrenia: neurocognitive correlates. J. Nerv. Ment. Dis..

[42] Kiritsis, P. Preserving the creative advantages of schizophrenia: a quantitative pretest-posttest study on the effects of cognitive remediation training on creativity. (Sofia University, 2018).

[43] Barlati S (2019). Factors associated with response and resistance to cognitive remediation in schizophrenia: a critical review. Front. Pharmacol..

[44] Seccomandi B, Tsapekos D, Newbery K, Wykes T, Cella M (2020). A systematic review of moderators of cognitive remediation response for people with schizophrenia. Schizophr. Res. Cogn.

[45] Wykes T (2012). Developing models of how cognitive improvements change functioning: mediation, moderation and moderated mediation. Schizophr. Res..

[46] Eack SM, Geile MFP, Greenwald DP, Hogarty SS, Keshavan MS (2011). Mechanisms of functional improvement in a 2 ­ year trial of cognitive enhancement therapy for early schizophrenia Mechanisms of functional improvement in a 2-year trial of cognitive enhancement therapy for early schizophrenia. Psychol. Med..

[47] Rispaud SG, Rose J, Kurtz MM (2016). The relationship between change in cognition and change in functional ability in schizophrenia during cognitive and psychosocial rehabilitation. Psychiatry Res..

[48] Fiszdon JM, Choi J, Goulet J, Bell MD (2008). Temporal relationship between change in cognition and change in functioning in schizophrenia. Schizophr. Res..

[49] Seccomandi B (2021). Can IQ moderate the response to cognitive remediation in people with schizophrenia?. J. Psychiatr. Res..

[50] Seccomandi B (2021). Exploring the role of age as a moderator of cognitive remediation for people with schizophrenia. Schizophr. Res..

[51] Kurtz MM, Wexler BE, Fujimoto M, Shagan DS, Seltzer JC (2008). Symptoms versus neurocognition as predictors of change in life skills in schizophrenia after outpatient rehabilitation. Schizophr. Res..

[52] Twamley EW, Burton CZ, Vella L (2011). Compensatory cognitive training for psychosis: who benefits? who stays in treatment?. Schizophr. Bull..

[53] Wykes T (2007). Cognitive remediation therapy (CRT) for young early onset patients with schizophrenia: an exploratory randomized controlled trial. Schizophr. Res..

[54] Twamley EW, Savla GN, Zurhellen CH, Heaton RK, Jeste V (2009). Cognitive training intervention for people with psychosis. Am. J. Psychiatr. Rehabil..

[55] Kurtz MM, Gagen E, Rocha NBF, Machado S, Penn DL (2016). Comprehensive treatments for social cognitive deficits in schizophrenia: A critical review and effect-size analysis of controlled studies. Clin. Psychol. Rev..

[56] Ahmed AO (2015). A randomized study of cognitive remediation for forensic and mental health patients with schizophrenia. J. Psychiatr. Res..

[57] Kalinowska S (2021). The association between lifestyle choices and schizophrenia symptoms. J. Clin. Med..

[58] Farreny A (2016). Baseline predictors for success following strategy-based cognitive remediation group training in schizophrenia. J. Nerv. Ment. Dis..

[59] Bell MD, Choi KH, Dyer C, Wexler BE (2014). Benefits of cognitive remediation and supported employment for schizophrenia patients with poor community functioning. Psychiatr. Serv..

[60] Cai C, Yu L, Rong L, Zhong H (2014). Effectiveness of humor intervention for patients with schizophrenia: a randomized controlled trial. J. Psychiatr. Res..

[61] American Psychiatric Association. *Diagnostic and Statistical Manual of Mental Disorders*. (American Psychiatric Association, 2013).

[62] Csernansky JG, Mahmoud R, Brenner R (2002). A comparison of risperidone and haloperidol for the prevention of relapse in patients with schizophrenia. N. Engl. J. Med..

[63] Faul F, Erdfeldel E, Lang A-G, Buchner A (2007). G*Power 3: a flexible statistical power analysis program for the social, behavioral, and biomedical. Behav. Res. Methods.

[64] Schretlen, D. *Modified Wisconsin Card Sorting Test professional manual*. (PAR, 2010).

[65] Wechsler, D. *WAIS-III Manual: Wechsler Adult Intelligence Scale-III*. (Psychological Corporation, 1997).

[66] Golden, C. J. *STROOP: Test de colores y palabras*. (2010).

[67] Brandt, J. & Benedict, R. *Hopkins Verbal Learning Test– Revised*. (Psychological Assessment Resources, 2001).

[68] Happé FG (1994). An advanced test of theory of mind: understanding of story characters’ thoughts and feelings by able autistic, mentally handicapped, and normal children and adults. J. Autism Dev. Disord..

[69] Johannesen JK, Lurie JB, Fiszdon JM, Bell MD (2013). The social attribution task-multiple choice (SAT-MC): a psychometric and equivalence study of an alternate form. ISRN Psychiatry.

[70] Bell M, Bryson G, Lysaker P (1997). Positive and negative affect recognition in schizophrenia: a comparison with substance abuse and normal control subjects. Psychiatry Res..

[71] Torrance, E. P. *The Torrance Tests of Creative Thinking — Norms-Technical Manual Research Edition—Verbal Tests, Forms A and B— Figural tests, Forms A and B*. (Personnel Press, 1966).

[72] Torrance, E. P. *Torrance Tests of Creative Thinking*. (Scholastic Testing Service. Inc., 2016).

[73] Garcia-Portilla MP (2013). Validation of a European Spanish-version of the University of California performance Skills Assessment (Sp-UPSA) in patients with schizophrenia and bipolar disorder. Schizophr. Res..

[74] Alonso J (2008). Desarrollo y validación de la versión corta de la Escala de Funcionamiento Social en esquizofrenia para su uso en la práctica clínica. Actas Esp. Psiquiatr..

[75] Gooding DC, Fonseca-Pedrero E, Pérez De Albéniz A, Ortuño-Sierra J, Paino M (2016). Adaptación española de la versión para adultos de la Escala de Placer Interpersonal Anticipatorio y Consumatorio. Rev. Psiquiatr. Salud Ment..

[76] Sanjuán Suárez P, Pérez García AM, Bermúdez Moreno J (2000). Escala de autoeficacia general: Datos psicométricos de la adaptación para población española. Psicothema.

[77] Kay SR, Fiszbein A, Opler LA (1987). The positive and negative syndrome scale (PANSS) for schizophrenia. Schizophr. Bull..

[78] Wallwork RS, Fortgang R, Hashimoto R, Weinberger DR, Dickinson D (2012). Searching for a consensus five-factor model of the Positive and Negative Syndrome Scale for schizophrenia. Schizophr. Res..

[79] Carpenter WT, Blanchard JJ, Kirkpatrick B (2016). New standards for negative symptom assessment. Schizophr. Bull..

[80] Kirkpatrick B, Fenton WS, Carpenter WT, Marder SR (2006). The NIMH-MATRICS consensus statement on negative symptoms. Schizophr. Bull.

[81] Kirkpatrick B (2011). The brief negative symptom scale: psychometric properties. Schizophr. Bull..

[82] Feixas G (2012). Escala de satisfacción con el tratamiento recibido (CRES-4): la versión en español. Rev. Psicoter..

[83] Del Ser T, Gonzalez-Montalvo JI, Martinez-Espinosa S, Delgado-Villapalos C, Bermejo F (1997). Estimation of premorbid intelligence in Spanish people with the word accentuation test and its application to the diagnosis of dementia. Brain Cogn..

[84] Nelson, H. E. & Willison, J. *National Adult Reading**Test (NART)*. (1991).

[85] Gomar JJ (2011). Validation of the Word Accentuation Test (TAP) as a means of estimating premorbid IQ in Spanish speakers. Schizophr. Res..

[86] Oldfield RC (1971). The assessment of handedness: the Edinburgh inventory. Neuropsychologia.

[87] Ojeda, N. & Peña, J. *REHACOP: programa de rehabilitación neuropsicológica en psicosis*. (Parima Digital, 2012).

[88] Efron, B. & Tibshirani, R. *An Introduction to the Bootstrap*. (Chapman & Hall, 1993).

[89] Cohen, J.*Statistical Power Analysis for the Behavioral Sciences*. (1988).

[90] Jöreskog, K. & Sörbom, D. LISREL 9.20 for Windows [Computer software]. (2015).

[91] Hu LT, Bentler PM (1999). Cutoff criteria for fit indexes in covariance structure analysis: conventional criteria versus new alternatives. Struct. Equ. Model..

