# Supplementary material for: Cognitive, creative, functional, and clinical symptom improvements in schizophrenia after an integrative cognitive remediation program: a randomized controlled trial
Source: NPJ Schizophr. 2021 Oct 28;7:52. doi: 10.1038/s41537-021-00181-0 (PMC8553761; doi:10.1038/s41537-021-00181-0)
Supplement: Supplementary file 1 — Supplementary Information [file 41537_2021_181_MOESM1_ESM.pdf]

Supplementary Table 1

Socio-demographic and clinical characteristics of the sample at baseline.

		REHACOP group (n = 42)		Active control group (n = 39)			
		Mean<br>n (%)	SD	Mean<br>n (%)	SD	*t/U/*X^2	*p*
Age (years)		41.88	10.11	42.13	10.88	0.106	.916
Education (years)		10.3	2.42	10.15	2.98	772.00	.654
Gender	Males	36 (85.7%)		30 (76.9%)		1.04	.309
	Females	6 (14.3%)		9 (23.1%)			
Handedness	Right-handed	33 (78.6%)		32 (82.1%)		3.051	.217
	Left-handed	0 (0%)		2 (5.1%)			
	Mixed-handed	9 (21.4%)		5 (12.8%)			
Age of onset (years)		24.07	5.92	23.21	7.43	712.00	.311
Previous hospitalizations		6.26	6.63	7.94	7.87	733.00	.414
Hospitalization status	Outpatients	22 (52.4%)		19 (48.7%)		0.109	.742
	Inpatients	20 (47.6%)		20 (51.3%)			
Medication dosage		514.70	298.98	482.84	196.61	805.00	.895
Premorbid IQ		95.62	10.02	93.97	9.99	706.00	.284

SD = Standard Deviation; *t* = *t*-test; *U* = Mann-Whitney *U*; X^2 = Chi-squared;

Medication dosage refers to chlorpromazine equivalent doses (mg/day).

Supplementary Table 2

Neurocognitive, creativity, and social cognitive performance in the REHACOP and Active Control groups at baseline and post treatment.

	REHACOP group (n = 42)		Active control group (n = 39)		Baseline comparison	
	Mean (95% CI)	SD	Mean (95% CI)	SD	*t/U*	*p*
**Neurocognition**						
CF						
Pre	0.06 (-0.17 to 0.29)	0.74	-0.06 (-0.39 to 0.25)	1.03	803.50	.883
Post	0.15 (-0.08 to 0.37)	0.71	-0.16 (-0.49 to 0.16)	1.07		
PS						
Pre	0.20 (-0.08 to 0.49)	0.88	-0.21 (-0.45 to 0.01)	0.71	2.30	.024
Post	0.38 (0.13 to 0.66)	0.83	-0.41 (-0.64 to -0.19)	0.70		
WM						
Pre	0.16 (-0.08 to 0.40)	0.82	-0.17 (-0.52 to 0.19)	1.15	673.00	.157
Post	0.31 (0.03 to 0.60)	0.97	-0.34 (-0.65 to -0.04)	0.93		
VM						
Pre	0.00 (-0.29 to 0.30)	0.96	-0.00 (-0.27 to 0.29)	0.93	816.50	.981
Post	0.28 (-0.02 to 0.60)	0.99	-0.31 (-0.55 to -0.06)	0.78		
Inhibition						
Pre	0.12 (-0.18 to 0.42)	0.98	-0.13 (-0.42 to 0.16)	0.88	701.00	.265
Post	0.34 (0.07 to 0.61)	0.89	-0.36 (-0.64 to -0.11)	0.85		
**Social cognition**						
ToM						
Pre	4.48 (3.76 to 5.17)	2.39	3.74 (2.92 to 4.56)	2.70	680.50	.187
Post	5.40 (4.90 to 5.93)	1.70	3.08 (2.49 to 3.67)	1.93		
SP						
Pre	9.38 (8.15 to 10.72)	4.07	8.69 (7.14 to 10.20)	4.86	733.00	.415

Post	10.60 (9.32 to 11.75)	3.95	10.69 (9.41 to 11.98)	4.18		
EP						
Pre	14.88 (13.66 to 16.10)	4.12	14.46 (13.11 to 15.74)	4.20	785.00	.747
Post	16.26 (15.30 to 17.15)	2.97	13.64 (12.39 to 14.97)	4.23		
**Creativity**						
Figural Creativity						
Pre	52.66 (46.57 to 58.91)	21.38	44.62 (39.99 to 49.83)	16.12	655.00	.121
Post	52.27 (44.97 to 60.54)	24.01	45.43 (41.03 to 49.68)	13.85		
Figural Creative Strengths						
Pre	2.40 (1.71 to 3.20)	2.39	2.72 (1.76 to 3.85)	3.39	0.49	.624
Post	2.36 (1.59 to 3.22)	2.62	1.64 (0.97 to 2.41)	2.29		
Verbal Creativity						
Pre	20.31 (17.33 to 23.68)	10.51	15.22 (12.90 to 17.57)	7.34	560.00	.014
Post	20.74 (17.11 to 24.33)	11.49	16.79 (13.86 to 19.73)	9.17		

CF = Cognitive Flexibility; PS = Processing Speed; WM = Working Memory; VM = Verbal Memory; ToM = Theory of Mind; SP = Social Perception; EP = Emotion Processing. CI = Confidence Interval; SD = Standard Deviation; *t* = *t*-test; *U* = Mann-Whitney *U*. CI was derived from the bootstrap analysis.

Supplementary Table 3

Clinical symptoms and functional outcome in the REHACOP and Active Control groups at baseline and post treatment.

	REHACOP group (n = 42)		Active control group (n = 39)		Baseline comparison	
	Mean (95% CI)	SD	Mean (95% CI)	SD	*t/U*	*p*
**Functional outcome**						
Functional competence						
Pre	63.44 (59.39 to 67.34)	13.88	60.68 (56.40 to 65.08)	13.56	0.90	.369
Post	75.31 (72.42 to 78.05)	9.34	63.07 (58.29 to 67.86)	15.25		
Social functioning*						
Pre	24.38 (22.87 to 25.97)	4.82	24.05 (21.71 to 26.37)	5.52	0.23	.816
Post	23.35 (21.77 to 25.03)	5.06	24.15 (22.00 to 26.46)	5.14		
Hedonic capacity*						
Pre	76.38 (71.85 to 80.75)	14.19	78.90 (71.67 to 85.99)	16.23	0.38	.706
Post	74.38 (70.62 to 78.32)	12.98	80.30 (72.90 to 87.60)	17.53		
General self-efficacy*						
Pre	60.35 (53.57 to 67.07)	21.44	58.15 (49.24 to 67.58)	19.77	0.61	.545
Post	61.14 (54.95 to 67.33)	19.09	62.15 (54.68 to 69.71)	17.69		
**Clinical symptoms**						
Negative						
Pre	28.81 (24.66 to 32.85)	14.11	35.02 (30.85 to 39.41)	13.59	-2.02	.047
Post	22.62 (18.17 to 27.04)	14.53	33.39 (29.15 to 37.95)	13.79		
Positive						
Pre	10.29 (9.10 to 11.57)	4.32	8.72 (7.66 to 9.69)	3.49	1.79	.078
Post	8.67 (7.64 to 9.70)	3.48	8.28 (7.19 to 9.46)	3.69		

Disorganization						
Pre	7.64 (6.77 to 8.58)	3.07	7.70 (6.91 to 8.46)	2.56	-0.09	.927
Post	6.74 (5.88 to 7.64)	2.90	7.58 (6.86 to 8.34)	2.36		
Excitement						
Pre	7.86 (6.76 to 9.16)	3.78	6.98 (6.03 to 8.05)	3.23	727.00	.378
Post	6.50 (5.67 to 7.46)	2.99	6.77 (5.81 to 7.94)	3.44		
Depression						
Pre	6.45 (5.79 to 7.15)	2.39	6.20 (5.51 to 6.94)	2.19	768.50	.629
Post	5.71 (4.98 to 6.48)	2.46	6.04 (5.39 to 6.80)	2.21		

CI = Confidence Interval; SD = Standard Deviation; *t* = *t*-test; *U* = Mann-Whitney *U*. CI was derived from the bootstrap analysis. *Sample size in these measures was n = 37 for the REHACOP group and n = 20 for the active control group.

Supplementary Table 4

Differences in change scores between the REHACOP and Active Control groups *after controlling for baseline scores.*

	REHACOP group (n = 42)		Active control group (n = 39)		ANCOVA for change scores		Effect size
	Mean change score (95% CI)	SE	Mean change score (95% CI)	SE	*F*	*p*	η_p^2
**Neurocognition**							
CF	0.11 (-0.10 to 0.35)	0.11	-0.12 (-0.37 to 0.12)	0.12	2.156	.150	0.027
PS	0.23 (0.09 to 0.37)	0.07	-0.25 (-0.40 to -0.10)	0.08	20.94	.001	0.212
WM	0.24 (-0.00 to 0.48)	0.13	-0.26 (-0.61 to 0.07)	0.17	6.99	.014	0.082
VM	0.28 (0.04 to 0.48)	0.11	-0.30 (-0.51 to -0.11)	0.10	17.35	.001	0.184
Inhibition	0.28 (0.06 to 0.53)	0.12	-0.31 (-0.59 to -0.03)	0.15	11.61	.003	0.130
**Social cognition**							
ToM	1.12 (0.56 to 1.71)	0.30	-0.88 (-1.43 to -0.42)	0.26	39.78	.001	0.338
SP	1.41 (0.16 to 2.75)	0.66	1.79 (0.55 to 2.92)	0.61	0.22	.645	0.003
EP	1.47 (0.66 to 2.35)	0.43	-0.91 (-1.92 to 0.13)	0.53	14.85	.001	0.160
**Creativity**							
Figural Creativity	1.25 (-4.12 to 7.49)	2.95	-0.95 (-5.54 to 3.97)	2.45	0.341	.537	0.004

Figural Creative Strengths	-0.12 (-0.74 to 0.56)	0.35	-1.00 (-1.61 to -0.38)	0.31	4.45	.033	0.054
Verbal Creativity	2.43 (-1.09 to 6.32)	1.85	-0.59 (-4.11 to 2.98)	1.83	1.59	.193	0.020
**Functional outcome**							
Functional competence	12.27 (9.59 to 14.97)	1.36	1.96 (-0.91 to 4.72)	1.41	32.55	.001	0.294
Social functioning*	-0.98 (-2.45 to 0.42)	0.72	0.02 (-1.78 to 1.54)	0.88	0.80	.379	0.015
Hedonic capacity*	-2.25 (-5.62 to 0.93)	1.62	1.86 (-2.95 to 6.99)	2.61	2.11	.194	0.038
General self-efficacy*	1.14 (-4.08 to 6.59)	2.67	3.33 (-3.75 to 9.94)	3.51	0.274	.582	0.005
**Clinical symptoms**							
Negative	-6.75 (-9.86 to -4.20)	1.45	-1.03 (-3.44 to 1.17)	1.18	9.297	.003	0.108
Positive	-1.41 (-2.19 to -0.54)	0.41	-0.66 (-1.52 to 0.11)	0.41	2.050	.149	0.026
Disorganization	-0.91 (-1.43 to -0.39)	0.27	-0.11 (-0.54 to 0.37)	0.23	5.618	.018	0.067
Excitement	-1.22 (-1.82 to -0.63)	0.30	-0.36 (-1.13 to 0.59)	0.44	2.946	.099	0.036
Depression	-0.70 (-1.35 to 0.03)	0.35	-0.20 (-0.85 to 0.42)	0.32	1.233	.257	0.016

CF = Cognitive Flexibility; PS = Processing Speed; WM = Working Memory; VM = Verbal Memory; ToM = Theory of Mind; SP = Social Perception; EP = Emotion Processing. CI = Confidence Interval; SE = Standard Error; ANCOVA, analysis of covariance; η_p^2 = partial eta

squared; Change scores = post-treatment score - pre-treatment score. Means for change scores are adjusted for the effect of the baseline score. CI and SE for change scores were derived from the bootstrap analysis. Significance levels were determined using F tests based on the bootstrap SE estimate for that comparison, rather than using a pooled SE estimate. *Sample size in these measures was n = 37 for the REHACOP group and n = 20 for the active control group.
