# Supplementary material for: Cognitive, creative, functional, and clinical symptom improvements in schizophrenia after an integrative cognitive remediation program: a randomized controlled trial
Source: NPJ Schizophr. 2021 Oct 28;7:52. doi: 10.1038/s41537-021-00181-0 (PMC8553761; doi:10.1038/s41537-021-00181-0)
Supplement: Supplementary file 2 — Reporting Summary [file 41537_2021_181_MOESM2_ESM.pdf]

nature research

Corresponding author(s): Javier Peña
Last updated by author(s): Jul 23, 2021

Reporting Summary

Nature Research wishes to improve the reproducibility of the work that we publish. This form provides structure for consistency and transparency in reporting. For further information on Nature Research policies, see our Editorial Policies and the Editorial Policy Checklist.

Statistics

For all statistical analyses, confirm that the following items are present in the figure legend, table legend, main text, or Methods section.

n/a	Confirmed	
☐	☒	The exact sample size (n) for each experimental group/condition, given as a discrete number and unit of measurement
☐	☒	A statement on whether measurements were taken from distinct samples or whether the same sample was measured repeatedly
☐	☒	The statistical test(s) used AND whether they are one- or two-sided<br>*Only common tests should be described solely by name; describe more complex techniques in the Methods section.*
☐	☒	A description of all covariates tested
☐	☒	A description of any assumptions or corrections, such as tests of normality and adjustment for multiple comparisons
☐	☒	A full description of the statistical parameters including central tendency (e.g. means) or other basic estimates (e.g. regression coefficient) AND variation (e.g. standard deviation) or associated estimates of uncertainty (e.g. confidence intervals)
☐	☒	For null hypothesis testing, the test statistic (e.g. F, t, r) with confidence intervals, effect sizes, degrees of freedom and P value noted<br>*Give P values as exact values whenever suitable.*
☒	☐	For Bayesian analysis, information on the choice of priors and Markov chain Monte Carlo settings
☒	☐	For hierarchical and complex designs, identification of the appropriate level for tests and full reporting of outcomes
☐	☒	Estimates of effect sizes (e.g. Cohen's d, Pearson's r), indicating how they were calculated

Our web collection on statistics for biologists contains articles on many of the points above.

Software and code

Policy information about availability of computer code

Data collection	n.a.
Data analysis	n.a.

For manuscripts utilizing custom algorithms or software that are central to the research but not yet described in published literature, software must be made available to editors and reviewers. We strongly encourage code deposition in a community repository (e.g. GitHub). See the Nature Research guidelines for submitting code & software for further information.

Data

Policy information about availability of data

All manuscripts must include a data availability statement. This statement should provide the following information, where applicable:

- Accession codes, unique identifiers, or web links for publicly available datasets
- A list of figures that have associated raw data
- A description of any restrictions on data availability

The data that support the findings of this study are available from the corresponding author on reasonable request. The data are not publicly available due to them containing information that could compromise research participant privacy or consent.

Field-specific reporting

Please select the one below that is the best fit for your research. If you are not sure, read the appropriate sections before making your selection.

☒ Life sciences ☐ Behavioural & social sciences ☐ Ecological, evolutionary & environmental sciences

For a reference copy of the document with all sections, see nature.com/documents/nr-reporting-summary-flat.pdf

Life sciences study design

All studies must disclose on these points even when the disclosure is negative.

Sample size	The sample consisted of 94 patients diagnosed with schizophrenia
Data exclusions	The exclusion criteria were as follows: a) clinical instability according to the criteria of Csernansky et al.61; b) relevant modifications in the antipsychotic treatment in the previous three months due to an increase in the severity of clinical symptoms; c) cognitive impairment caused by another disease; d) diagnosis of an active Major Affective Disorder; e) being in another specific cognitive remediation program; and f) Substance Use Disorder (DSM-5)60 within the three months before screening.
Replication	n.a.
Randomization	The study design was a parallel-group randomized trial, forming part of a larger project that has been later expanded to include an additional arm (a group receiving intense physical exercise). The cognitive remediation arm has been completed, while the physical exercise arm is ongoing. Therefore, in the present study only the cognitive remediation group and the active control group were included. The participants were randomly assigned to each group, this being done via an online computer-generated randomization system (http://www.randomization.org). Randomization was performed by a Study Coordinator who was not involved in the assessment, study interventions, or statistical analyses.
Blinding	All raters were blind to the experimental treatment condition and had no other role in the study (they had no clinical responsibility for the participants during the completion of the study and were not involved in administering the cognitive remediation or occupational activities) that could undermine the trial blinding. Moreover, participants were instructed not to mention what type of treatment they would receive or had received during evaluations

Reporting for specific materials, systems and methods

We require information from authors about some types of materials, experimental systems and methods used in many studies. Here, indicate whether each material, system or method listed is relevant to your study. If you are not sure if a list item applies to your research, read the appropriate section before selecting a response.

Materials & experimental systems

n/a	Involved in the study	
☒	☐	Antibodies
☒	☐	Eukaryotic cell lines
☒	☐	Palaeontology and archaeology
☒	☐	Animals and other organisms
☐	☒	Human research participants
☒	☐	Clinical data
☒	☐	Dual use research of concern

Methods

n/a	Involved in the study	
☒	☐	ChIP-seq
☒	☐	Flow cytometry
☒	☐	MRI-based neuroimaging

Human research participants

Policy information about studies involving human research participants

Population characteristics	The sample consisted of 94 patients diagnosed with schizophrenia who were recruited from the Psychiatric Hospital of Álava and the Mental Health Network in Álava (Spain). All patients had been diagnosed with schizophrenia according to the Diagnostic and Statistical Manual of Mental Disorders, Fifth Edition (DSM-5). The final sample included 81 patients (66 males and 15 females. The mean age of the sample was 41.88 (SD = 10.11) years old.
Recruitment	Participants were recruited between 2017 and 2021 at the Psychiatric Hospital of Álava and the Mental Health Network in Álava (Spain).
Ethics oversight	The Clinical Research Ethics Committees of the Autonomous Region of the Basque Country (CEIC-E) in Spain (PI2017044).

Note that full information on the approval of the study protocol must also be provided in the manuscript.
